# Natural and long-term preservable anticoagulant property of SiO_2_ and TiO_2_ bilayer films

**DOI:** 10.3389/fbioe.2025.1578099

**Published:** 2025-05-19

**Authors:** Xiangqin Liu, Xiao Chen, Hongrui Jiang, Zikun He, Hong Sun, Qiongjian Huang, Ansha Zhao, Nan Huang, Ping Yang, Jiang Chen

**Affiliations:** ^1^ The Department of Ophthalmology, Sichuan Provincial People’s Hospital, University of Electronic Science and Technology of China, Chengdu, China; ^2^ Department of Laboratory Medicine, Sichuan Academy of Medical Sciences and Sichuan Provincial People's Hospital, University of Electronic Science and Technology, Chengdu, China; ^3^ Department of Stomatology, The Institute of Oral Science, Longgang Otorhinolaryngology Hospital of Shenzhen, Shenzhen, China; ^4^ Key Laboratory for Advanced Technologies of Materials, Institute of Biomaterials and Surface Engineering, Ministry of Education, Southwest Jiaotong University, Chengdu, China; ^5^ Department of Cardiology, Sichuan Provincial People’s Hospital, University of Electronic Science and Technology of China, Chengdu, China; ^6^ Office of Scientific Research, Chongqing Industry Polytechnic College, Chongqing, China; ^7^ Clinical Immunology Translational Medicine Key Laboratory of Sichuan Province, Sichuan Provincial People’s Hospital, University of Electronic Science and Technology of China, Chengdu, China

**Keywords:** SiO_2_ and TiO_2_ bilayer films, long-term anticogulant property, long-term superhydrophilicity, platelet, fibrinogen

## Abstract

**Introduction:**

Titanium dioxide (TiO2) films have been widely studied as blood-contacting materials, but their positively charged surface and low density of surface hydroxyl (-OH) groups result in poor intrinsic anticoagulant properties. Furthermore, TiO_2_ surfaces readily adsorb carbon-containing contaminants from the environment, causing a rapid decline in anticoagulant performance during storage. Thus, improving TiO_2_'s intrinsic anticoagulant properties and extending its shelf-life remain challenging.

**Methods:**

We fabricated a bilayer film by depositing a ∼40 nm silica (SiO2) overlayer onto TiO_2_ using unbalanced magnetron sputtering. Surface properties (hydrophilicity, surface charge, and contaminant adsorption) and anticoagulant performance (platelet adhesion after storage) of the resulting SiO_2_/TiO_2_ bilayer were characterized.

**Results:**

The SiO_2_/TiO_2_ bilayer exhibited long-lasting hydrophilicity, a net negative surface charge, minimal adsorption of carbonaceous contaminants, and a high surface -OH group content. These characteristics are attributed to the formation of interfacial Si–O–Ti bonds, which in turn led to significantly enhanced anticoagulant properties. Notably, after 15 weeks of storage, platelet surface coverage on the bilayer was less than 30% of that on a TiO_2_-only film, indicating greatly improved long-term hemocompatibility.

**Discussion:**

By maintaining a hydrophilic, clean surface with abundant surface -OH groups, the SiO_2_/TiO_2_ bilayer achieved superior intrinsic anticoagulant performance that was preserved over long-term storage. This bilayer approach addresses key limitations of TiO_2_, suggesting that SiO_2_/TiO_2_ coatings are a promising alternative to pure TiO_2_ films for blood-contacting devices.

## 1 Introduction

Titanium dioxide (TiO_2_) films have been widely used as surface modification materials for blood-contacting devices (Nan et al., 1998), such as vascular stents (Yang et al., 2019; Cui et al., 2021), due to their relatively good hemocompatibility (Shao et al., 2010), corrosion resistance (Shan et al., 2008), and biosafety (Zhang et al., 2020). However, the anticoagulant properties of TiO_2_ films still need to be further enhanced. Since the 1990s, many modification methods for enhancing the anticoagulant properties of TiO_2_ films have been reported. One of the typical strategies is to modify anticoagulant biomolecules or load drugs on the TiO_2_ surface (Li et al., 2018; Huang et al., 2014; Fan et al., 2016). However, introducing biomolecules or drugs brings new problems, such as the complex and poorly reproducible preparation processes and the long-term stability of modified biomolecules *in vivo* (Weng et al., 2011; Yang et al., 2017). On the other hand, changing the structures or chemical features of the TiO_2_ films to enhance its anticoagulant properties has not stopped. The mechanism of those strategies can be attributed to: (1) increasing hydrophilicity (Xu et al., 2017). The hydrophilic surface can effectively reduce blood protein and platelet adhesion. (2) Loading negative charge (Zhao et al., 2014). It can reduce the adhesion of negatively charged proteins in the blood through electrostatic repulsion. (3) Increasing −OH content (Yi et al., 2018). −OH can effectively reduce the degree of protein denaturation during the adsorption process. However, the long-term preservation of the anticoagulant properties of TiO_2_ films under routine storage has received less attention.

Since 2008, the aging issue of the bioactivity of TiO_2_ has started to attract attention (Att et al., 2009a; Att et al., 2009b). Clean TiO_2_, which is superhydrophilic and significantly positively charged, can promote protein and cell adhesion and growth. However, with the increase of storage time, TiO_2_ continuously adsorbs carbon-containing adsorbates from the air, leading to the loss of hydrophilicity and the change of surface charge from positive to negative, leading to the decline of protein and cell adhesion (Hori et al., 2010; Iwasa et al., 2010).

The gradual transformation of the naturally bioactive surface of TiO_2_ into a biologically inert surface when stored in the air became an essential insight for using Ti materials in dental and orthopedic applications (Ueno et al., 2010; Hirota et al., 2020). At the same time, this insight enlightens us that: (1) when TiO_2_ is used as a blood contact material, its anticoagulant ability is necessarily affected by the increase of carbon-containing adsorbates during the storage. (2) The new TiO_2_, with the hydrophilic but positively charged surface, which promotes the negative charged plasma protein adhesion (Barbir et al., 2021), may be detrimental to inhibiting thrombus generation. (3) After the storage, the negatively charged TiO_2_ surface, which is hydrophobic and contains procoagulant -CH_3_ groups from the carbon-containing adsorbates (Sivaraman and Latour, 2010a; Sivaraman and Latour, 2010b), may also be detrimental to inhibiting thrombus generation.

Starting in 2014, we have reported a series on the photo-induced anticoagulant properties of TiO_2_: after UV irradiation, by enhancing the hydrophilicity and oxidation of surface carbon-containing adsorbates, TiO_2_ films obtain excellent anticoagulant properties (Chen et al., 2014; Chen et al., 2015). However, this photo-induced anticoagulant property still diminishes with the increase in storage time (Liao et al., 2017). Therefore, long-term storage may be a common challenge for all types of Ti/TiO_2_-based blood contact devices since medical devices are subject to lengthy storage, transportation, and distribution processes before use.

We noticed that the anticoagulant properties of UV-treated TiO_2_ (TiO_2_-UV) are lost simultaneously with its photo-induced hydrophilic properties. Therefore, can the method to extend the storage time of TiO_2_ photophilic similarly extend the preservation time of its anticoagulant properties?

It has been reported that the construction of a surface consisting of a mixture of SiO_2_ and TiO_2_ by sol-gel method is an effective method for obtaining long-term superhydrophilicity (Guan and Yin, 2005). Meanwhile, the SiO_2_ and TiO_2_ mixed surface may be rich in -OH groups (Miyashita et al., 2001). However, the interface between SiO_2_ and TiO_2_ could form the Si-O-Ti bond, which has a positive electrostatic charge (Guan et al., 2003). The exposure of Si-O-Ti bond to the surface will be detrimental to the material’s anticoagulation. In this context, we envision the construction of bilayer films with SiO_2_ in the upper layer and TiO_2_ in the lower layer. This bilayer film could embed the positive Si-O-Ti bond at the interface, and the SiO_2_ surface is then likely to be negatively charged due to charge balance (Kim et al., 2011). This bilayer strategy requires the preparation of sufficiently thin and dense SiO_2_ films. Unbalance magnetron sputtering can produce dense TiO_2_ and SiO_2_ films with excellent mechanical properties on devices with complex surface topography, and the film thickness can be precisely controlled by the sputtering time and other process modulations (Buranawong et al., 2012; Seifarth et al., 1998).

In this study, we prepared TiO_2_ and SiO_2_ films by the unbalance magnetron sputtering. We prepared SiO_2_ films with different thicknesses on TiO_2_ films to obtain the SiO_2_ and TiO_2_ bilayer films. The bilayer films with SiO_2_ layer thicknesses of about 40 nm showed the best hydrophilic and anticoagulant properties. Then this optimal SiO_2_ and TiO_2_ bilayer film was evaluated in detail on the surface physical and chemical properties. Furthermore, the bilayer films’ natural and long-term hydrophilicity and long-term anticoagulant ability were systematically evaluated. Our results showed that the SiO_2_&TiO_2_ bilayer film had desirable natural and long-term superhydrophilicity and anticoagulant properties, which might be closely related to the inhibition of adsorption of carbon-containing adsorbates by the unique surfaces properties.

## 2 Materials and methods

### 2.1 Preparation of the SiO_2_ and TiO_2_ bilayer films

**FIGURE 1:**
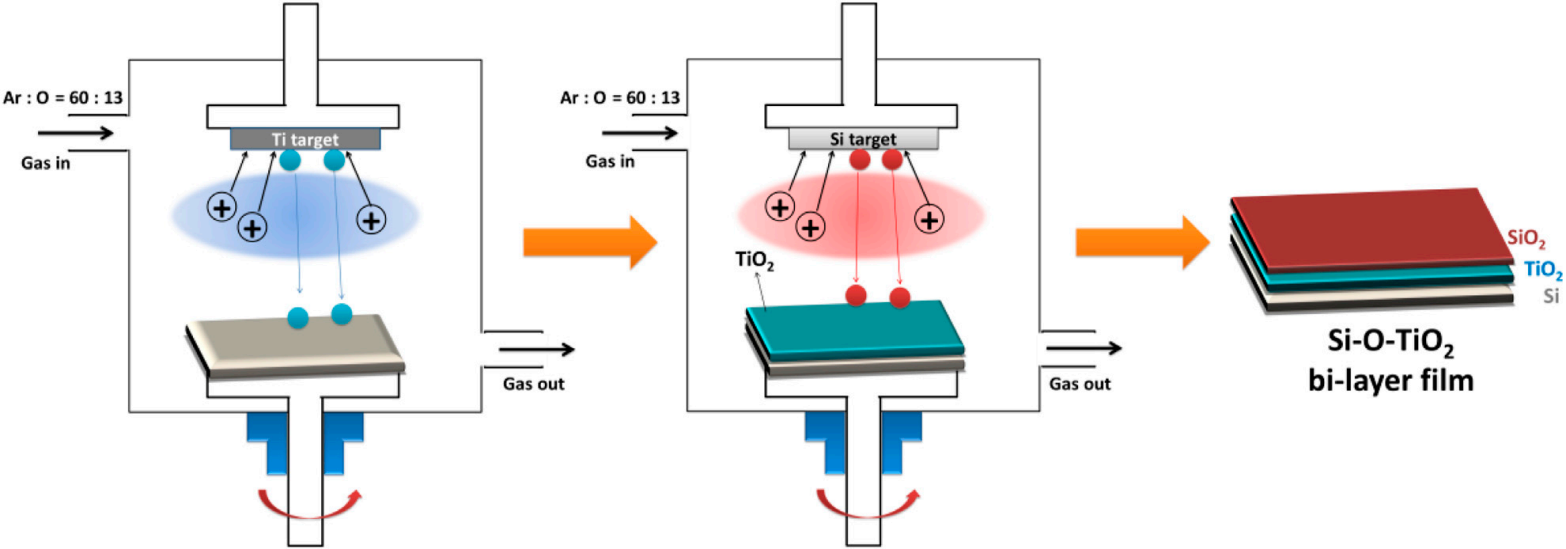
Schematic diagram of the preparation process of SiO_2_ and TiO_2_ bilayer film.

As shown in Figure 1, the TiO_2_ film was first prepared on a silicon substrate using the unbalance magnetron sputtering deposition system (UBMS450,Chinese Academy of Sciences, China), and then, in the same vacuum chamber, it was further covered with SiO_2_ film to obtain the SiO_2_ and TiO_2_ bilayer film. The detailed preparation parameters are shown in the Table 1.

**TABLE 1:** Experimental parameters of deposition of the TiO_2_ films and SiO_2_ films.

Parameters	Values (For TiO_2_ films)	Values (For SiO_2_ films)
Ar/O_2_ flow	60 sccm/13 sccm	60 sccm/13 sccm
Target distance	105 mm	85 mm
direct current	3 A	3 A
Bias	0 V	0 V
Deposition time	15 min	15 s, 30 s, or 60 s
Temperature (initial/final)	15/105°C	N/A

### 2.2 UV irradiation treatment

The samples were UV irradiated for 1 h by a lithography machine (URE-2000, Chinese Academy of Sciences, China). The UV light intensity was 16 mW/cm^2^, and the UV wavelength was 365 nm.

### 2.3 Material characterization

A surface profiler (Ambios XP-2, Ambios, Santa Cruz, CA) was employed to determine the thickness of the films. A drop shape analysis system (DSA 100, Krüss, Germany) was used to examine the hydrophilicity of the films by the sessile drop method (5-µL droplet).

The structures of TiO_2_ films were determined by X-ray diffraction (XRD) (X’Pert Pro MPD, Philips, Holland) using a copper target at a glancing angle of 0.5°. An atomic force microscope (AFM) (SPI 3800; NSK, Japan) was used in tapping mode to detect the roughness of the samples.

X-ray photoelectron spectroscopy (XPS, XSAM800, Kratos Ltd., United KIngdom) was used to detect the surface chemical features of the films. The instrument was equipped with a monochromatic Al Kα (1,486.6 eV) X-ray source operated at 12 kV × 15 mA at a pressure of 2 × 10^−10^ mbar. The C 1s peak at 284.8 eV was used as a reference for charge correction. The surface information of the samples were measured directly using XPS without any cleaning. The subsurface Information of the samples were measured using XPS after ion-beam sputtering.

EST111 Static Charge Meter (EST Electro-Static Test, Co. Ltd., China) was used to detect the surface charge of samples (18 × 18 mm^2^).

### 2.4 Storage of samples

Samples were stored in the air for 1 day to 15 weeks to study the long-term preservation of surface properties and biological properties of the samples. The samples were placed in 24-well plates, covered by cling film to avoid adhesion of dust in the surface air, and stored in a class 10,000 clean room.

### 2.5 Platelet adhesion assay and P-selectin staining assay

The platelets adhesion test was detailly described elsewhere (Yang et al., 2020). Scanning electron microscope (SEM; Quanta 200, FEI, Holland) was used to observe the shape of adhered platelet. The optical microscope (DM4000M, Leica, Germany) was used to calculate the platelet surface coverage (PSC).

P-selectin was determined by indirect immunochemistry to examine the activation level of adhered platelets. Firstly, added 50 mL PRP on the samples and incubated at 37°C for 60 min. Then, washed the samples for 3 times with PBS. After that, each sample was covered with 20 µL of FITC-labeled antieCD62P (1:100) (MCA796GA, Serotec Co.). After incubating at 37°C for 60 min, washing samples for thress times, drying by N_2_ flow. Then the stained samples were observed under an inverted fluorescence microscope (IX51, Olympus, Japan).

### 2.6 Fibrinogen adsorption and its conformational change

Fresh platelet-poor plasma (PPP) was obtained by centrifuging venous blood at 3000 rpm for 15 min. Then, 40 µL of PPP was dropped on the TiO_2_ samples (7 × 7 mm^2^). Afterward, samples were incubated at 37°C for 1 h. Then, we use the immunochemistry method to determine the relative quantification of fibrinogen adsorption and conformational change.

#### 2.6.1 Fibrinogen adsorption test

Firstly, the samples were incubated with PPP and were rinsed thoroughly with PBS. Then the samples were blocked with 1 wt% bovine serum albumin (BSA) at 37°C for 1 h. Subsequently, rewashed the samples and added 20 µL of HRP(Horseradish Peroxidase)-labeled mouse-anti-human fibrinogen monoclonal antibody (first antibody, diluted 1:200 in PBS). Then, the samples were incubated at 37°C for 1 h before adding 70 µL of chromogenic substrate TMB solution (diluted 1:4 in PBS) to the samples’ surface for color reaction. After reacting for 15 min, 50 µL of 1 M H_2_SO_4_ was added to stop the color reaction. Afterward, 70 µL of the solutions on the sample’s surface was removed to a 96-well plate to be examined by a microplate reader at 450 nm. The ratio of adsorbed fibrinogen was calculated according to the calibration curve.

#### 2.6.2 Fibrinogen conformational change test

The detection of fibrinogen conformational changes is broadly similar to the fibrinogen adsorption assay. The differences are: (1) the first antibody used is a mouse-anti-human γ-fibrinogen monoclonal antibody; (2) after the introduction of the first antibody and sufficient washing, a further second antibody was introduced: HRP-labeled goat-anti-mouse polyclonal antibody (second antibody, diluted 1:200 in PBS). The relative quantification of the exposure of fibrinogen’s γ chain was calculated according to the calibration curve.

### 2.7 Data analysis and statistics

Three parallel samples (n = 3) were used for all experiments. Statistical significance between sample groups was assessed using SPSS 11.5 software with one-way ANOVA and LSD *post hoc* test. Differences between samples were considered statistically different when P < 0.05.

## 3 Results and discussion

### 3.1 Effect of SiO_2_ film thickness on hydrophilicity and anticoagulant properties of SiO_2_ and TiO_2_ bilayer films

**FIGURE 2:**
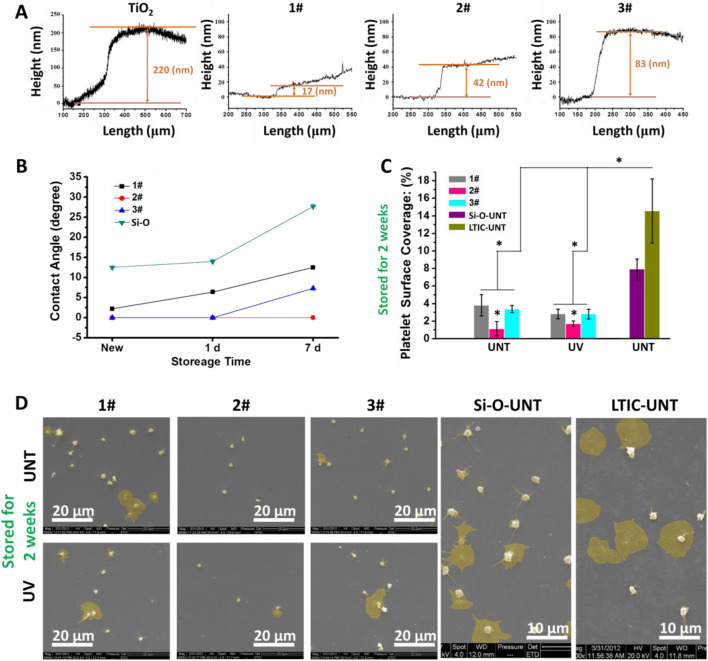
**(A)** Characterization of film thickness, **(B)** hydrophilicity of film after 7 days of storage in air, **(C)** statistics of platelet adhesion area ratio on film surface after 7 days of storage in air. (Data expressed as mean ± optical microscope, and analyzed using the one-way ANOVA and LSD *post hoc* test, *p < 0.05). **(D)** SEM photographs of adhered platelets.

As shown in Figure 2A, the TiO_2_ film was obtained by magnetron sputtering with a thickness of about 220 nm. Then the SiO_2_ films were deposited on the TiO_2_ film to obtain the SiO_2_&TiO_2_ bilayer films. By altering the film deposition time, SiO_2_&TiO_2_ bilayer films with SiO_2_ films with thicknesses of about 17 nm, 42 nm, and 83 nm were prepared, which were named 1#, 2#, and 3#, respectively. In addition, SiO_2_ films were deposited on Si as control samples.

As shown in Figure 2B, the newly fabricated 1#, 2#, and 3# were superhydrophilic surfaces (water contact angle <5°) (Padilla and Carey, 2014), among which the water contact angle of 2# and 3# were even 0°. At the same time, the SiO_2_ films directly deposited on Si had a water contact angle of about 12.5° and did not exhibit superhydrophilicity. After 1 day of storage, the water contact angle of 1# rose to 6.4° and lost superhydrophilicity, while 2# and 3# still maintained superhydrophilicity. When stored for 7 days, the water contact angles of SiO_2_, 1#, and 3# were about 7.3°, 12.5°, and 27.7° respectively, while the water contact angle of 2# remained at 0° and showed a superhydrophilicity.

Figure 2C showed the results of the platelet surface coverage (PSC, calculated from optical microscope photos) of samples. In the absence of UV treatment, the PSC values on 1#, 2#, and 3# were 3.8%, 1.1% and 3.3%, respectively, significantly lower than those on the positive control LTIC(7.9%) and SiO_2_ films (14.6%). The PSC value of 2# was lower than that of 1# and 3#. Meanwhile, the UV treatment showed no significant effect on the anticoagulant properties of the bilayer films (1#, 2#, 3#).

Figure 2D showed that many activated and spreading platelets were observed on the surfaces of the positive controls (SiO_2_ and LTIC). In 1# and 3#, most platelets adhere to the sample’s surface in a spherical shape, and only a few platelets undergo spreading, indicating the activation of platelets was inhibited (Chesnutt and Han, 2013). Moreover, in 2#, only a tiny amount of platelets were found to undergo spreading. Combining the hydrophilicity, platelet adhesion, and SEM results, we elected 2# for further study.

### 3.2 Structural and surface physical-chemical state analysis of SiO_2_&TiO_2_ bilayer films

**FIGURE 3:**
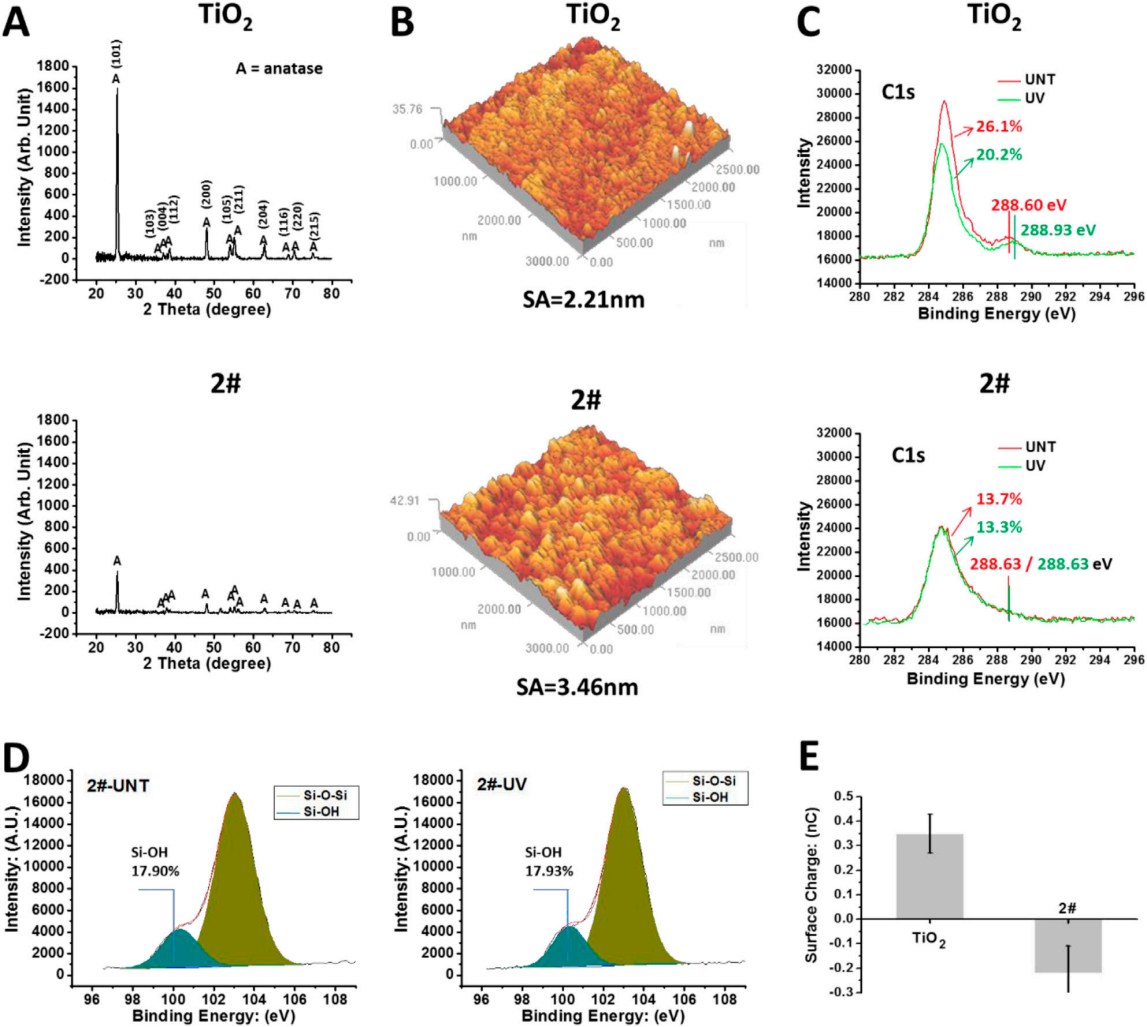
**(A)** XRD results of TiO_2_ with SiO_2_ and TiO_2_ bilayer films. **(B)** AFM results of the samples. **(C)** After 4 weeks of storage, XPS results of the sample surface: C1s high-resolution spectrum. **(D)** After 4 weeks of storage, the Si2p high-resolution spectrum on the surface of sample 2#. **(E)** The surface charge of the samples’ surface after 4 weeks of storage.

As shown in Figure 3A, the XRD results indicated that the TiO_2_ obtained by magnetron sputtering is anatase crystalline type (Tekdir et al., 2021). After being covered by the SiO_2_ film, the XRD results of sample 2# showed a substantial decrease in the intensity of the TiO_2_ signal. Meanwhile, SiO_2_ signal was not observed, which could be related to the SiO_2_ layer being thin or not highly crystallized. As shown in Figure 3B, the roughness of the TiO_2_ film was 2.2 nm, while the roughness of sample 2# slightly increased to 3.4 nm.

Then, we tested the chemical state of the samples’ surface using XPS without any cleaning. As shown in Figure 3C, after 4 weeks of storage, the shoulder peak around 288.6 eV, a characteristic peak of oxygen-containing hydrocarbon adsorbate (Ti-O-C) (Baek et al., 2013), was observed on the TiO_2_-UNT surface. This peak shifted toward 288.93 eV after UV irradiation, implying the oxidation of oxygen-containing hydrocarbon adsorbates due to the photocatalytic oxidation effect (Mattsson and Österlund, 2010), resulting in the generation of Ti-O-C=O residues (Xing et al., 2014). In contrast, on the surface of sample 2#, UV treatment did not affect the binding energy of the shoulder peak around 288.6 eV, which implied that 2# does not have the photocatalytic oxidation ability. In addition, the percentage of C element in the TiO_2_ surface was 26.1%, which decreased to 20.2% after UV irradiation. The decrease of C element on the surface of TiO_2_ after UV treatment was closely related to the photocatalytic self-cleaning effect of the TiO_2_ (Guan, 2005).

In comparison, the percentage of C elements on the surface of 2# was 13.7% and 13.3% before/after UV irradiation, indicating the 2# sample did not own the photocatalytic self-cleaning effect. However, the total amount of C elements on the 2# sample surface, about 13%, was significantly lower than that on the TiO_2_ surface (26.1%), even after UV self-cleaning (20.2%), indicating a significantly low carbon-containing adsorbate on the bilayer surface after 4 weeks of storage (Liao et al., 2021). This low amount of carbon-containing adsorbates on the surface of 2# might be beneficial and preserve the material surface’s inherent properties, such as hydrophilic properties (Antonello et al., 2014).

As shown in Figure 3D, the Si2p high-resolution spectrum of XPS showed that the surface of 2#, after 4 weeks of storage, contained a large amount of Si-OH (Paparazzo et al., 1992), and the UV treatment did not affect its Si-OH content. The large amount of -OH might bring high hydrophilicity and a negative charge to the surface of 2# (Houmard et al., 2011). As shown in Figure 3E, after 4 weeks of storage, the TiO_2_ surface had a positive charge (about 0.35 nC), while the 2# surface had a negative charge (about −0.22 nC).

In summary, SiO_2_ and TiO_2_ bilayer films had a low contamination rate, abundant hydroxyl groups, and negative charge, all of which might be beneficial to reduce coagulation reactions of the bilayer films in blood and ensure their anticoagulant properties for long-term storage in the air.

### 3.3 Analysis of the chemical state at the interface between SiO_2_ and TiO_2_ for SiO_2_ and TiO_2_ bilayer films

**FIGURE 4:**
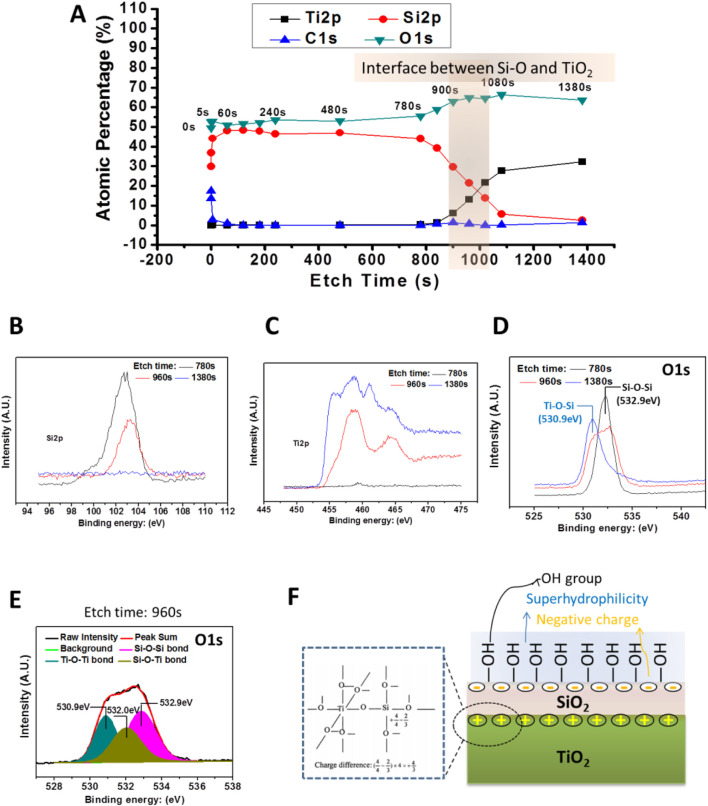
**(A)** XPS results of the bilayer film (2#) etching. **(B,C)** High-resolution patterns of Si2p and Ti2p peaks of the bilayer film were detected by XPS after different, etch times. **(D)** The high-resolution spectrum of O1s peaks of the bilayer films were detected by XPS after different, etch times. **(E)** O1s high-resolution spectrum of 2# after etching for 960 s. **(F)** Schematic diagram of 2# samples at the interface between TiO_2_ and SiO_2_ films and the possible physiochemical characteristics of the bilayer film’s surface.

As shown in Figure 4A, the characteristic elemental signals of the 2# surface and the subsurface were detected using XPS at argon plasma etching (etching times (TE) = 0 s, 5 s, and 60 s). The results showed a high Si content on the surface and subsurface (TE = 0–60 s) of the 2# sample, and no Ti element was found. It indicated that SiO_2_ layer achieved a continuous and complete coverage of the TiO_2_ layer. When the TE extended to 840 s, the signal of the Si element started to fall, while the signal of the Ti element started to rise. When the TE = 1080 s, the Si element signal drops to within 5%, while the Ti element percentage rises to more than 25%. Obviously, when TE = 840–1080 s, the signal from the interface between SiO_2_ and TiO_2_ was collected.

Furthermore, by analyzing Figure 4A, it could be seen that TE = 960 s, the signal was from the SiO_2_ and TiO_2_ interface, while TE = 780 s and TE = 1380 s, the signal could be come from the SiO_2_ film and TiO_2_ film near the interface, respectively. Therefore, we analyze the high-resolution spectra of Si2p, Ti2p, and O1s when TE = 780 s, 960 s, and 1380 s. As shown in Figures 4B,C, for TE = 780 s, a strong Si peak is detected, while the Ti peak signal is very weak, and the opposite was true for the TE = 1380 s surface, where only the Ti signal was detected, while without the Si peak. In contrast, when TE = 960 s, both Si and Ti peaks were detected, and these peaks were lower than the Si peak at TE = 780 s and the Ti peak at TE = 960 s, respectively. These results verify that when TE = 780 s, 960 s, and 1380 s, signals were from SiO_2_, SiO_2_/TiO_2_ interface, and TiO_2_, respectively.

As shown in Figure 4D, the O1s high-resolution spectrum showed that when TE = 780 s, the peak at 532.9 eV could be attributed to the Si-O-Si bond, while the TE = 1380 s, the peak at 530.9 eV might come from the Ti-O-Ti bond (Gao and Wachs, 1999). However, it was interesting that when TE = 960 s, a broad peak appeared at 530.9–533.0 eV. Further analysis of this peak revealed (Figure 4E) that the O1s peak at TE = 780 s could be divided into three peaks. Besides the Si-O-Si bond and Ti-O-Ti bond mentioned earlier, an additional peak at 520.0 eV appeared, which could be attributed to the Si-O-Ti bond (Zhang et al., 2004). This result indicated that during the preparation of SiO_2_ films, Si atoms enter into the TiO_2_ film, forming a Si-O-Ti bond. As illustrated in Figure 4F, the Si-O-Ti bond formation leads to the charge imbalance so that a positive-rich charge might be formed at the interface between SiO_2_ and TiO_2_ (Guan et al., 2003; Kim et al., 2011), while a negative charge was formed on the outer surface of the SiO_2_ layer according to the charge balance principle (Permpoon et al., 2008). At the same time, the charge imbalance at the interface induced the formation of a large number of -OH groups. This might be why the SiO_2_ and TiO_2_ bilayer film’s surface was negatively charged and rich in -OH groups (Zhang et al., 2019).

### 3.4 Hydrophilicity testing of SiO_2_ and TiO_2_ bilayer films after long-term of storage

**FIGURE 5:**
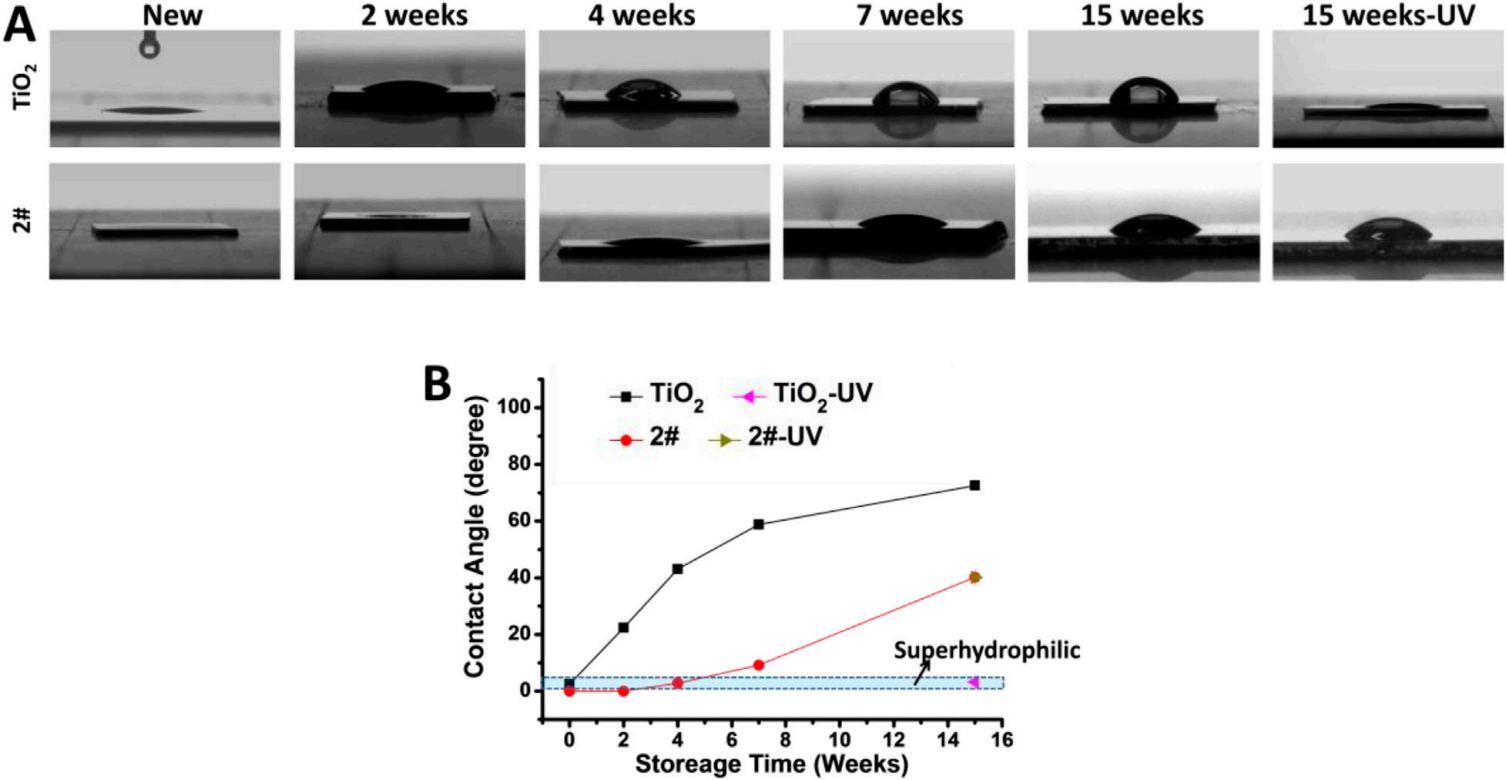
Water contact angle test results: **(A)** photo, **(B)** statistics.

As shown in Figures 5A,B, the water contact angle of the new TiO_2_ film was 2.1°, which exhibited superhydrophilicity. While the water contact angle of the newly made 2# sample is 0, indicating that its hydrophilicity was more potent than that of the new TiO_2_ film. After 2 weeks of storage in air, the water contact angle of TiO_2_ increased to 22.4°, while the water contact angle of 2# was still 0°. After 4 weeks of storage, the water contact angle of TiO_2_ continued to rise to 42.5°, while the water contact angle of 2# was only 2.6°, still maintaining superhydrophilicity. After 7 weeks of storage, the water contact angle of TiO_2_ was 58.5°, while that of 2# was only 9.6°. Therefore, the natural superhydrophilicity of 2# seemed could maintain in the air for more than 4 weeks. Finally, when the storage time came to 15 weeks, the TiO_2_ water contact angle increased to 71°, while the 2# water contact angle was only 38.6°. The above results show that the bilayer film had better hydrophilic properties than TiO_2_, and its hydrophilic properties could be preserved in the air longer than TiO_2_. Further, after UV irradiation treatment of the 15-week-stored TiO_2_, its water contact angle decreased to 3.2°, exhibiting photo-induced superhydrophilicity, which could be related to the abundant of–OH in its surface (Houmard et al., 2011). In contrast, the hydrophilicity of sample 2# did not change after UV irradiation. This result indicated that it did not possess photo-induced hydrophilic properties, which could be related to the absence of the photocatalytic activity of the bilayer films (Wang et al., 1997).

### 3.5 Anticoagulant property analysis of SiO_2_ and TiO_2_ bilayer films after long-term of storage

**FIGURE 6:**
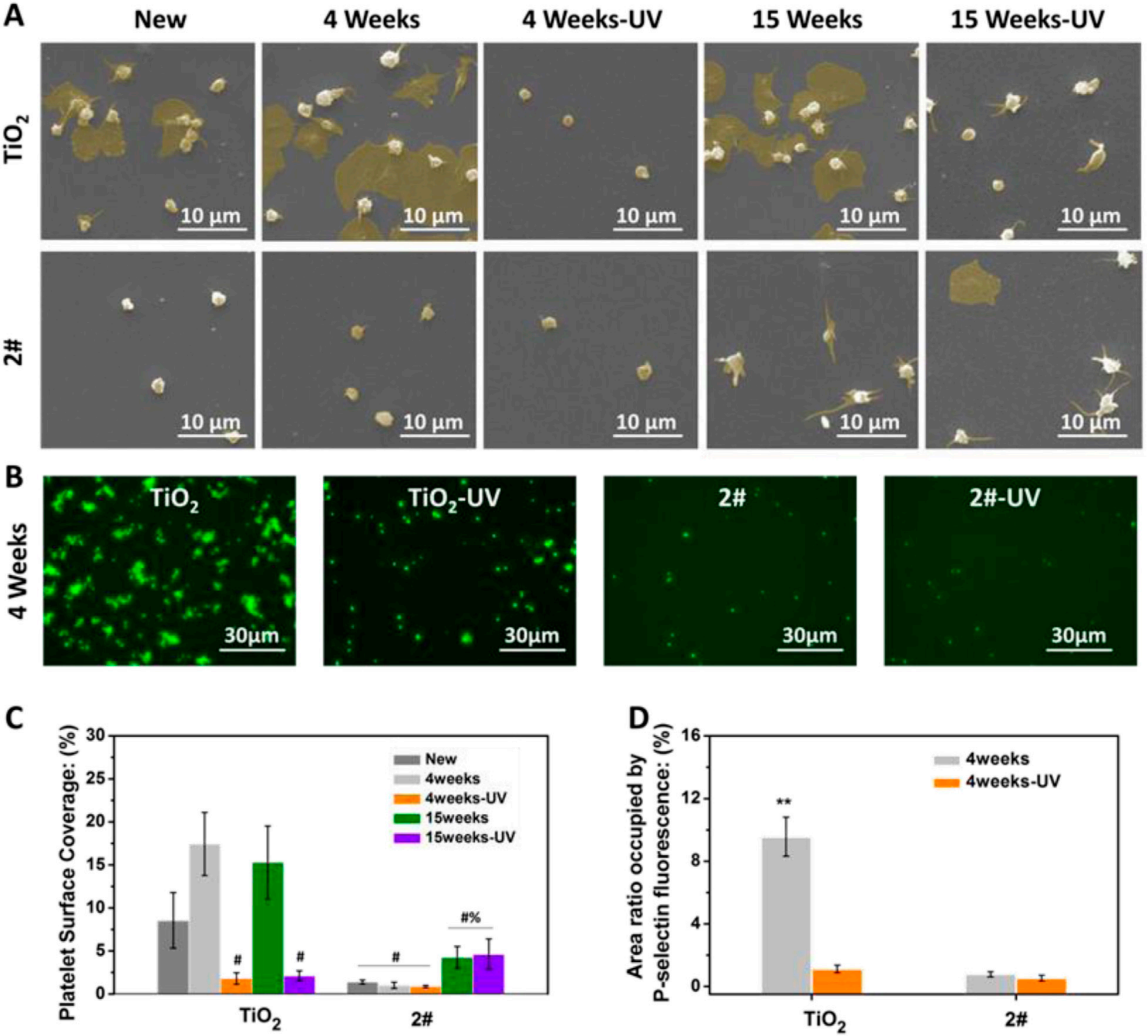
**(A)** SEM photographs of platelets, **(B)** platelet surface coverage (PSC) (# indicating the significant difference (*p < 0.05*) between samples and the positive controls (untreated TiO_2_ films), % indicating the significant difference (*p < 0.05*) between samples and the negative controls (UV-treated TiO_2_ films)), **(C)** P-selectin staining photographs, **(D)** P-selectin statistical results. (**p < 0.01) (Data expressed as mean ± optical microscope or fluorescence microscope, and analyzed using the one-way ANOVA and LSD *post hoc* test).

As shown in Figures 6A,C, many activated and spreading platelets were found on the freshly prepared, stored for 4 weeks, and stored for 15 weeks TiO_2_ surfaces. The platelet surface coverage (PSC, calculated from optical microscope photos) of the above samples was 8.2%, 17.4%, and 15.2%, respectively. After UV treatment, the PSC of TiO_2_ stored for 4 and 15 weeks significantly decreased to 1.8% and 1.4% due to the photo-induced anticoagulant effect of TiO_2_. Moreover, the platelet morphology on the TiO_2_-UV surface was predominantly spherical, indicating the platelets’ activiation was suppresed (Chesnutt and Han, 2013).

For sample 2#, only a small amount of spherical platelet adhesion was found on the surface of the freshly prepared sample, and its PSC was about 1.0%. Only a small amount of spherical platelet adhesion was found for 4 weeks of stored 2#, no matter with or without UV irradiation. The PSCs of 2#-UNT and 2#-UV were about 0.8% and 0.7%, showing an excellent anticoagulant property. On the surface of samples stored for 15 weeks, no matter with or without UV irradiation, most platelets on 2# extended pseudopods and individual platelets underwent spreading. The PSCs of 2#-UNT and 2#-UV were about 4.2% and 4.6%.

The above results indicated that the new and the 4 weeks stored 2# bilayer films possess anticoagulant properties comparable to, or even better than, UV-irradiated TiO_2_ films. After 15 weeks of storage, the anticoagulant properties of 2# decreased but were still remarkable. This result fully illustrated that the bilayer film strategy could be expected to provide long-term, excellent anticoagulant properties.

As shown in Figures 6B,D, P-selectin staining was performed on the platelets adhered to the samples after 4 weeks of storage. Many P-selectin-positive platelets were found on the TiO_2_ surface, accounting for a surface area ratio of 9.5%, indicating a large number of platelet activation (Blann and Lip, 1997). On the TiO_2_-UV surface, P-selectin-positive cells were substantially reduced, only in 1.1% of the surface area. In contrast, on the 2# and 2#-UV surfaces, the area ratio of P-selectin positive cells was 0.8% and 0.5%, which is less than the value of TiO_2_-UV. This result indicated that 2# could inhibit platelet activation better than TiO_2_-UV. This result further illustrated the excellent natural anticoagulant property of SiO_2_ and TiO_2_ bilayer films and the long-term preservation of the anticoagulant properties.

### 3.6 Possible mechanism of the anticoagulant property of SiO_2_ and TiO_2_ bilayer films

**FIGURE 7:**
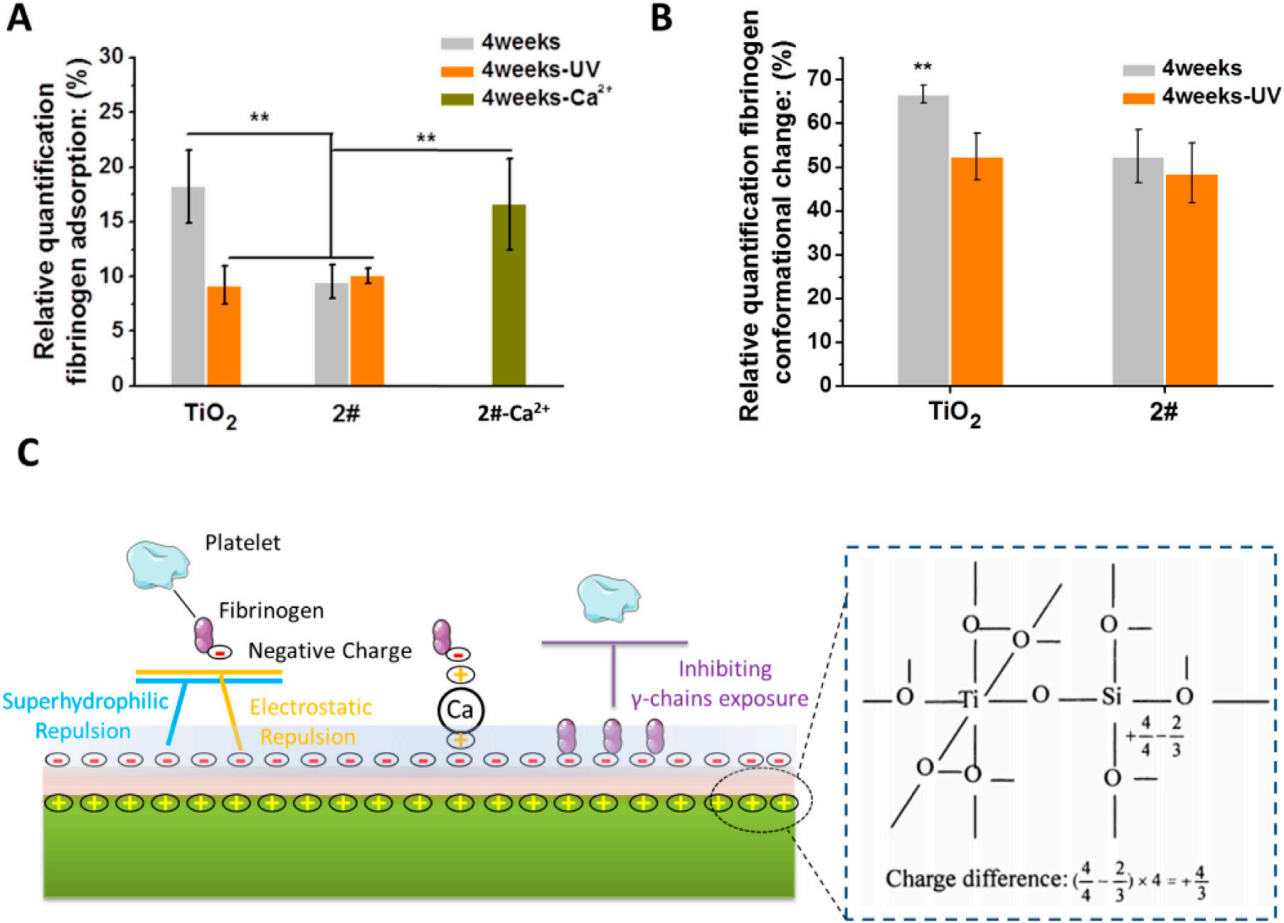
**(A)** Fibrinogen adsorption. **(B)** Fibrinogen conformational changes. **(C)** Possible anticoagulation mechanism of SiO_2_ and TiO_2_ bilayer films.

Fibrinogen is a negatively charged plasma protein with a molecular weight of 330 kD (Fujikawa et al., 2001; Price et al., 2001), and it is the coagulation factor I (Sørensen et al., 2012). Adsorption of fibrinogen to the material’s surface is a fundamental cause of coagulation. Therefore, we further investigated the adhesion behavior of fibrinogen in order to study the anticoagulation mechanism of SiO_2_ and TiO_2_ films. The samples were stored for 4 weeks before the test. As shown in Figure 7A, the fibrinogen adsorption on TiO_2_ without and with UV treatment was about 18.6% and 8.7%, respectively. As for 2# without and with UV irradiation, the fibrinogen adsorption values were 8.8% and 9.1%, respectively. This result indicated that 2# bilayer films had the same strong ability to inhibit fibrinogen adsorption as TiO_2_-UV. In particular, the ability of the 2# sample to inhibit fibrinogen adsorption was abolished when it was pretreated with Ca^2+^ for 12 h. This indicated that the electrostatic repulsion worked during the negatively charged 2# surface inhibited the adsorption of negatively charged fibrinogen in the blood (Iwasa et al., 2010).

Fibrinogen denaturation is a more direct cause of blood coagulation than fibrinogen adhesion (Sivaraman and Latour, 2010a). Therefore, we performed the fibrinogen conformational change experiments. As shown in Figure 7B, the 2# sample had a natural ability to inhibit fibrinogen denaturation close to that of TiO_2_-UV. The superhydrophilic (Li et al., 2020), low carbon-containing adsorbates (Liao et al., 2021) adsorption and -OH group-rich nature (Rodrigues et al., 2006) of 2# played a crucial role in its anticoagulant property.

As shown in Figure 7C, we briefly summarized the possible mechanisms of the long-term anticoagulant properties of SiO_2_ and TiO_2_ bilayer films. The formation of Ti-O-Si bond at the interface between SiO_2_ and TiO_2_ induced the loading of positive charge at the interface (Guan et al., 2003; Kim et al., 2011), thus resulting in the surface of the bilayer film becoming negative charging (Permpoon et al., 2008), superhydrophilic (Houmard et al., 2011), rich in -OH groups (Zhang et al., 2019), and low in carbon-containing adsorbates adsorption (Houmard et al., 2011). These unique physical-chemical properties reduce fibrinogen adsorption on 2#, most likely through hydrophilic and electrostatic repulsion. In addition, the bilayer films inhibited the denaturation of fibrinogen, which might be achieved by the large amount of -OH group that could maintain the natural conformational of fibrinogen (Rodrigues et al., 2006). More importantly, the anticoagulant properties of this SiO_2_ and TiO_2_ bilayer film lasted up to 15 weeks when stored in air, probably due to its -OH group-rich and long-term hydrophilic property, which resulted in slow contamination of its surface with carbon-containing adsorbates (Houmard et al., 2011), and thus prolonged preservation of its anticoagulant properties.

Recently, Zhao et al. from Sichuan University reported an anticoagulant coating with hidden positive charges, which concealed the positive charges through PEG and prevented the adsorbed factor XII from initiating subsequent coagulation by the fixation effect of positive charges on factor XII and the hydrophilic effect of PEG (Ji et al., 2024). Our research has significant similarities with theirs, but we are the first to achieve the above design through inorganic materials. We believe that this study has theoretical reference significance for the subsequent design of fixed-hydrophilic anticoagulant coatings.

## 4 Conclusion

The SiO_2_ and TiO_2_ bilayer films were prepared by the unbalance magnetron sputtering technique. The surface of this film exhibited the following features: (1) long-lasting superhydrophilicity for more than 4 weeks, (2) high content of -OH, (3) negative charging, and (4) low adsorption rate of carbon-containing absorbates. The above features might be closely related to forming a positively charged Si-O-Ti bond at the interface between the SiO_2_ and TiO_2_ layers. Due to their unique surface physicochemical properties, SiO_2_ and TiO_2_ bilayer films could effectively inhibit the adsorption and activation of fibrinogen in the blood, thus exhibiting excellent anticoagulant properties and could be stored in the air for a long time. This study provided a new idea to solve the difficulty of long-term preservation of the anticoagulant properties of TiO_2_ films. This strategy of bilayer films was expected to provide a theoretical reference for developing new multifunctional TiO_2_-based films.

## Data Availability

The original contributions presented in the study are included in the article/supplementary material, further inquiries can be directed to the corresponding authors.
